# New Insights into the Microbial Diversity of Cake Layer in Yttria Composite Ceramic Tubular Membrane in an Anaerobic Membrane Bioreactor (AnMBR)

**DOI:** 10.3390/membranes11020108

**Published:** 2021-02-03

**Authors:** Rathmalgodage Thejani Nilusha, Yuansong Wei

**Affiliations:** 1 State Key Joint Laboratory of Environmental Simulation and Pollution Control, Research Center for Eco-Environmental Sciences, Chinese Academy of Sciences, Beijing 100085, China; 2 Environment Technology Section, Industrial Technology Institute, 363, Bauddhaloka Mawatha, Colombo 07 00700, Sri Lanka; nthejani@yahoo.com or; 3 Department of Water Pollution Control Technology, Research Center for Eco-Environmental Sciences, Chinese Academy of Sciences, Beijing 100085, China; 4 University of Chinese Academy of Sciences, Beijing 100049, China; 5 Institute of Energy, Jiangxi Academy of Sciences, Nanchang 330029, China

**Keywords:** membrane bioreactor, ceramic tubular membrane, cake layer, bacteria, archaea

## Abstract

Cake layer formation is an inevitable challenge in membrane bioreactor (MBR) operation. The investigations on the cake layer microbial community are essential to control biofouling. This work studied the bacterial and archaeal communities in the cake layer, the anaerobic sludge, and the membrane cleaning solutions of anaerobic membrane bioreactor (AnMBR) with yttria-based ceramic tubular membrane by polymerase chain reaction (PCR) amplification of 16S rRNA genes. The cake layer resistance was 69% of the total membrane resistance. Proteins and soluble microbial by-products (SMPs) were the dominant foulants in the cake layer. The pioneering archaeal and bacteria in the cake layer were mostly similar to those in the anaerobic bulk sludge. The dominant biofouling bacteria were *Proteobacteria*, *Bacteroidetes*, *Firmicutes*, and *Chloroflexi* and the dominant archaeal were *Methanosaetacea* and *Methanobacteriacea* at family level. This finding may help to develop antifouling membranes for AnMBR treating domestic wastewater.

## 1. Introduction

Membrane bioreactor (MBR) technology is a promising technology for wastewater treatment and reuse [1]. Membranes can be coupled with either aerobic or anaerobic biological treatment processes. Anaerobic membrane bioreactors (AnMBR) provide more benefits compared to aerobic MBR. The costs of aeration and sludge handling in anaerobic treatment are considerably lesser than aerobic MBR [2]. Due to these unique benefits, AnMBR is attracting growing interest in both research and practical applications [3].

Meanwhile, ceramic membrane applications in MBR are achieving rapid progress attributed to their advantageous properties over widely applied polymeric membranes [4,5]. As an example, Ghyoot and Verstraete revealed that a commercial ceramic microfiltration (MF) membrane can reach 200–250 L/(m^2^·h) (LMH), which was 10-fold higher than the flux achieved with a polymer ultrafiltration (UF) membrane [6]. Moreover, ceramic membranes inherently owe low fouling propensity, chemical, and thermal stability, etc. [7]. AnMBR coupled with ceramic membranes (AnCMBR) has been previously studied in numerous studies investigating performance, and fouling, etc. [8,9,10,11,12].

Membrane fouling in AnCMBR is still a major bottleneck limiting its sustainable operation [13]. Generally, membrane fouling is the undesirable deposition of colloids, solutes, and accumulations of microorganisms and cell debris on membrane surfaces [14,15]. Cake layer formation on the membrane surface by organic and inorganic particles or biomaterial is the major contributor of the fouling in MBRs [16]. The accumulation of biomaterials on the membrane surface is named biofouling [17]. Biofouling is the most harmful and challenging to control [18]. Biofouling can be due to microbial colonization of membrane surfaces or deposition of bio foulants present within the bulk biomass [19,20]. Microbial participation on biofilm formation is dependent on the membrane material, module type, wastewater type and treatment temperature and type [21,22]. Considering novel ceramic membrane materials, the improved microstructure obtained by yttria (Y_2_O_3_) impregnation had a significant effect on enzyme loading yield and activity. This indicates the potential of this surface modification method and of these metal-supported ceramic membranes in enzyme immobilization [23]. Thus, yttria stabilized ceramic membrane show less biofouling, which reduces the cake layer resistance due to biofouling.

Identifying the key cake layer forming microbial species helps us to design and develop new membrane materials with biofouling resistivity [24]. Ceramic membrane biofouling has always been ignored and poorly demonstrated [25]. Only a few studies on microbial community attributed to cake layer in ceramic membrane processes have been reported [26]. Tubular membranes inherit comparative resistance for fouling due to the cross-flow velocity (CFV) over other membrane modules i.e., hollow fiber, flat sheet, etc. since their cake layer formation is disturbed by CFV [27]. Most interestingly, the yttria-based ceramic tubular membrane microbial fouling has not yet been elucidated in spite of aforesaid special biofouling reduction ability of yttria. Thus, the main objective of this work was understanding the microbial community in yttria-based tubular ceramic membrane fouling cake layer in AnMBR treating domestic wastewater at the ambient conditions. 16s rRNA based identification method was used for this investigation. To our present knowledge, this work is one of the primary studies discovering the cake layer microbial diversity in yttria composite ceramic tubular membrane. The findings of this study are vital for developing antifouling membranes for MBR in the future.

## 2. Materials and Methods

### 2.1. Description of MBR

A continuous stirred tank reactor (CSTR) made of plexiglass with an effective working volume of 15 L was used in this work and it was similar to our previous work [11,12]. The tubular membrane was made of ceramic, Yttria, Zirconia with a nominal pore size of 0.1 μm, and a total area of 0.11 m^2^ (HeFei ShiJie Membrane Engineering Co. Ltd., Hefei, China). A peristaltic pump (BT100-1L, Longer, YZ1515x Pump, Baoding, China) was used to feed synthetic domestic wastewater (The composition is given in our previous work [11] into the reactor and mixed liquor from the reactor was fed in to the external side stream membrane unit using a diaphragm pump (DP-35 diaphragm pump, Xin Xishan industries Co. Ltd., Shanghai, China) and retentate was returned to the reactor while permeate was collected in a tank. The membrane was externally installed allowing inside out filtration. The CFV inside the membrane was set at 2.5 m/s. This reactor was operated at the ambient temperature (31.2 ± 2.7 °C). The membrane module was operated in relaxation and recirculation modes including 5 min relaxation and 55 min operation. Permeate backwashing was conducted once a day at 60 s/day throughout the operation based on our previous work [12]. A programmable logic controller (PLC) system (LAB VIEW, PLC, Siemens AG, Frankfurt, Germany) was used for automatic operation. The bioreactor was operated at a hydraulic retention time (HRT) of 48 h and three varying solids retention time (SRT) s of 100, 50, 25 days at the ambient temperature. The reactor operational conditions and performance were well discussed in our previous publication [11].Operational transmembrane pressure (TMP) was established at 87 kPa with 54 L/(m^2^·h) as initial and sustainable fluxes. TMP was measured with a pressure transducer and controlled by the valves at the exit of the membrane unit, manually. The schematic diagram of the reactor set up is presented in Figure S1, which was also presented in our previous publications [11,12].

### 2.2. Cake Layer Sampling and Membrane Chemical Cleaning

The externally installed tubular membrane was dismantled from the reactor after 150 days of AnCMBR operation as the flux reduced over 50% of the original flux (16 L/(m^2^·h)). Firstly, the slightly appeared, cake layer was carefully and immediately collected by scraping it with a toothpick and spatula. The oxygen contact time with the cake layer is negligible to make a sufficient change in the microbial community as sampling was done immediately. Then the collected cake layer sample was refrigerated at −20 °C until the extraction of DNA.

The chemical cleaning sequence included (1) permeate cleaning then soaked in pure water for 8 h, (2) cleaning with NaOCl at effective Cl^−^ concentration of 500 ppm followed by soaking in pure water for 8 h, (3) cleaning with 500 ppm citric acid solution then soaked in pure water for 8 h.

### 2.3. Microbial Community Analysis

The samples for the microbial community analysis were collected from the seeds, anaerobic sludge at different stages (Day 45, Day 90 and Day 150), cake layer and the cleaning solutions. The genomic DNA of the microbial community in the collected samples was extracted by Fast DNA^®^ SPIN kit (MP Biomedicals, Solon, OH, USA). Bacterial community was evaluated by PCR amplification of 16S rRNA genes using the 515F/806R primers. For archaeal community Silva _Arch349F-Arch806R primers were used. Sequencing was conducted at the Sangon Co., Ltd. (Shanghai, China) sequencing center using pair-end Illumina sequencing (Illumina Inc., San Diego, CA, USA). The raw data were processed to obtain clean sequences on the free online platform of Majorbio Cloud Platform (www.majorbio.com (accessed on 1 January 2021)) of Shanghai Majorbio Bio-pharm Technology Co., Ltd., Shanghai, China using the project No. MJ20191010008-MJ-M-20191127012.This procedure and analysis were in accordance with [28].

### 2.4. Excitation-Emission Matrix (EEM) Fluorescence Spectroscopy

Major biopolymers present in the cleaning solutions were investigated by the three-dimensional excitation emission fluorescence (3D-EEM) analysis as biopolymers present in the cleaning solutions can be interlinked with the pioneering bacterial and archaeal communities in the cake layer. All samples for this analysis were measured for UV_250_ absorbance and diluted 50 times based on absorbance values. A fluorescence spectrophotometer (F-7000, Hitachi, Tokyo, Japan) was used for obtaining EEM spectra in the emission (EM) wavelength range of 220 and 550 nm excitation (EX) wavelength from 200 nm to 400 nm. Excitation and emission slits were set at 5 nm with a scanning speed of 12,000 nm min^−1^. Photomultiplier tube (PMT)) voltage was set to 700 V. Observed peaks were identified based on [29].

### 2.5. Cake Layer Resistance Calculation

Equation (1) is used to analyze the membrane filtration resistance according to Darcy law,

(1) $$R_{t}=\frac{\Delta P}{\mu J}$$

where, *J* is the permeate flux (m^3^·m^−2^·s^−1^), Δ*P* is the trans-membrane pressure (TMP) (Pa), μ is the viscosity of the permeate (Pa·s), *R_t_* is the total membrane filtration resistance (m^−1^) [30].

Equation (2) gives the calculation for cake layer resistance which is given as the reversible fouling resistance.

(2) $$R_{t}=R_{m}+R_{r}+R_{ir}$$

where *R_t_*, total membrane resistance, *R_m_* is the intrinsic membrane filtration resistance (m^−1^), *R_r_* is the reversible fouling resistance (m^−1^) and *R_ir_* is the irreversible fouling resistance (m^−1^).

*R_r_* is the resistance that can be removed by physical membrane cleaning, whereas *R_ir_* is the resistance that can be removed by chemical membrane cleaning. *R_t_* value was obtained by calculating the final flux of the membrane system at the end of the operation. *R_m_* was determined by measuring the deionized water flux with an unused membrane before employing the system. *R_m_ + R_ir_* was measured by physical cleaning of the membrane module with tap water to remove all the observable cake layer from its surface, and then flux measured with deionized water. Then, *R_ir_* value can be obtained by calculating the difference between *R_m_* and *R_ir_ + R_m_.* After getting the resistance values *R_t_, R_m_*, and *R_ir_, R_r_* can be calculated using Equation (2) according to [31,32,33].

## 3. Results and Discussions

### 3.1. AnCMBR Performance and Microbial Community Evolution

This study is the membrane cleaning and biofouling community investigation of our previous work [11]. There the major process performance parameters and the microbial community shifts were evaluated with the change of Solid Retention Times (SRT), 100 days, 50 days and 25 days at the ambient temperature. Accordingly, microbial community has showed significant shifts based on SRT. As described there, both bacterial and archaeal community diversities were higher at short SRT. There was a higher specific production of SMPs and extracellular polymeric substances (EPSs) at 25 days SRT compared to 50 days and 100 days SRT [11].

### 3.2. Cake Layer Resistance and Composition

The cake layer resistance and its contribution to total fouling resistance as a percentage from the total resistance was calculated using aforementioned Equations (1) and (2). Table 1 shows the calculated values for *R_t_*, *R_m_*, *R_r_* and *R_ir_*.

Accordingly, the cake layer (*R_r_*) has contributed 69.72% to the total membrane resistance with continuous once a day backwashing as the membrane fouling control strategy. Therefore, cake layer fouling had played a significant role in this study. The cake layer resistance at different MBR studies are presented in the Table 2. Accordingly in most studies PVDF membranes have shown over 80% of cake layer resistance from the total membrane resistance [33,34]. Ceramic flat sheet membranes have also shown over 80% of cake layer resistance as the major fouling resistance [9,35]. However, this ceramic membrane shows less than 70% cake layer resistance, which might be attributed to the membrane material, type of wastewater, operational mode, and membrane backwashing. The membrane used in this study was used in our previous work [11]. After cleaning it was re-installed for this experiment. Therefore, the intrinsic resistant value was one quarter of the total membrane resistance, which is comparatively higher. Further studies are needed regarding the cake layer resistance of different ceramic membrane modules.

**Figure 1:**
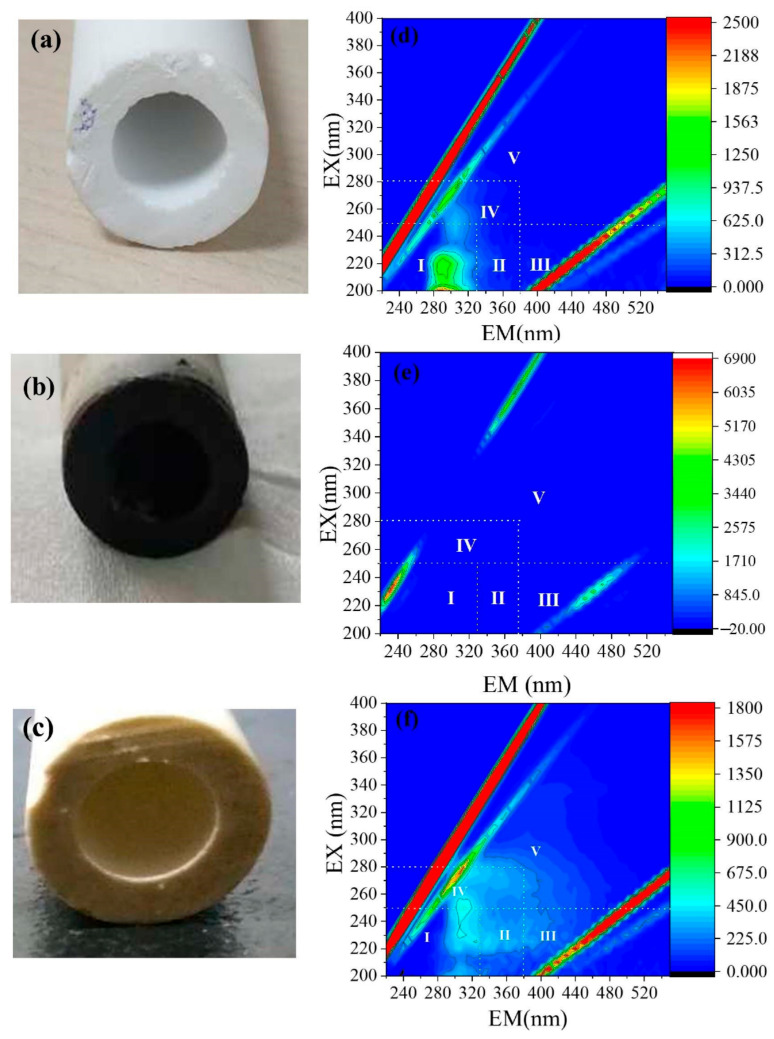
The evolution of the physical appearance of the ceramic tubular membrane and the biopolymer composition of membrane cleaning solutions (**a**) Virgin membrane (**b**) fouled membrane (**c**) cleaned membrane (**d**) permeate cleaning solution (**e**) NaOCl cleaning solution (**f**) citric acid cleaning solution.

The 3D-EEM fluorescence spectroscopy analysis of membrane cleaning solutions could present information on the organic components in the cake layer. According to the Figure 1, the permeate cleaning solutions showed the characteristic peaks of Region I and Region IV substances at Ex220/Em 290 and Ex 270/Em295, respectively which are ascribed to protein like substances and soluble microbial by products like substances (SMPs). The NaOCl cleaning solution showed the absence of any apparent peaks. This absence of the peaks in NaOCl cleaning solution was well described in our previous publication [12]. The citric acid cleaning solution also indicated the presence of Region I and Region IV substances at Ex230/Em305 and Ex 275.83/Em 306.08 respectively indicating the presence of protein like substances and SMPs. This implied that proteins and SMPs secreted by microbes are the major foulants which might easily attached to the ceramic tubular membrane.

### 3.3. Identification of Key Bacteria and Archaea in the Cake Layer

#### 3.3.1. Bacteria in Phylum Level in the Cake Layer

The cake layer bacterial community abundance is presented in the Table 3. Accordingly, the pioneering bacteria phyla in the cake layer were *Proteobacteria* accounting for 23% of the total bacteria abundance. *Bacteroidetes* (18%), *Firmicutes* (12%), *Chloroflexi* (18%) were subsequently dominant. The other phyla in minority over 1% abundance included *Thermotogae* (5.49%), *Spirochaetes* (3.93%), *Euryarchaeota* (1.8%), *Actinobacteria* (1.0%). The gamma *Proteobacteria* belonging to phylum *Proteobacteria* secrete extracellular polymeric substances (EPS), making them easier to adhere to the biofilm and promote biofilm formation [37]. *Bacteroidetes* are carbohydrate degraders, capable of EPS production contributing to fouling [38]. This might be the reason for their high abundance in the cake layer. Further, *Firmicutes* has the ability to accelerate biofouling in AnMBR and was commonly detected in fouling layers [15]. Also, *Firmicutes* secrete extracellular enzymes [39]. The contribution of *Bacteroidetes* and *Proteobacteria* for fouling in the present study comply with the more presence of proteinases biopolymers and soluble microbial by-products in the cleaning solutions. *Chloroflexi,* frequently identified as another dominant bacterial phylum involved in the cake layer, was also observed at high relative abundance (18%) in this study. *Chloroflexi* are filamentous bacteria and the filaments of these bacteria might adhere to and penetrate between membranes and foulants and aggravate membrane fouling. In contrast, Miura et al. indicated that *Chloroflexi* may alleviate biofouling in AnMBR due to reduced carbohydrate-rich SMPs or EPS accumulation in the reactor [40]. In this study, further foulant analysis such as EPS and total organic carbon (TOC) were not conducted in the cake layer because the formed cake layer (Figure 1b) throughout the operation was considerably low and it has been totally utilized for the bacterial and archaeal community investigations. However, more thorough investigations on the major foulants are required in future biofouling investigations such as EPS, SMP, TOC, etc.

#### 3.3.2. Top Archaea in Family Level in Cake Layer

There is limited information on the role of the archaeal community in biofilm formation in MBRs [41]. For the better interpretation of the archaea community developed in the cake layer, family level investigations were performed as shown in Table 4. The most dominant archaea families were *Methanosaetacea* (43%), *Methanobacteriaceae* (22%), *Methanomicrobiales* (14.2%), *Methanosarcinaceae* (8%), *Methanomassillicoccaceae* (8%).These are key archaeal families involve in anaerobic digestion [42]. In line with this study, Aslam et al. (2018) revealed the participation of *Methanosaetacea* family in biofilm formation in granular activated carbon samples in AnCMBR study [20]. The study conducted by Calderon et al., 2011 also showed presence of *Methanosaetacea* and *Methanobacteriaceae* in tubular ultrafiltration membranes made of fluoride polyvinylidene (PVDF) treating urban wastewater in a pilot scale up flow anaerobic sludge blanket (UASB) [43]. Another study further confirmed that biofilm community compose of *Methanobacteria* and *Methanomicrobia* in an anoxic/aerobic submerged biofilter system [41]. Further, Yue et al. reported that the *Methanosarcinaceae* family preferred to attach to the ceramic membranes [9]. These similarities of archaeal participation in biofilm formation might be as these are typical archaeal families in anaerobic digestion.

#### 3.3.3. Bacterial Community in Genus Level in the Cake Layer

**Figure 2:**
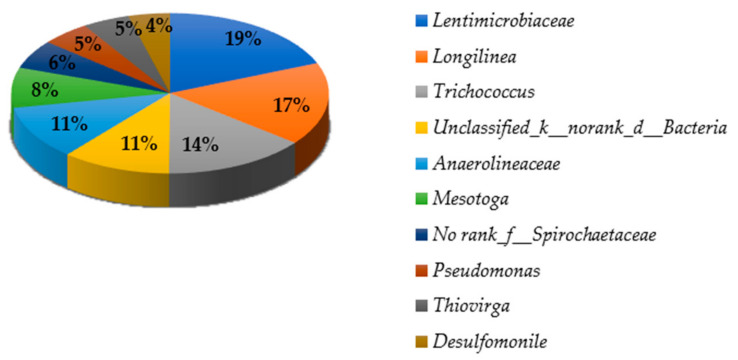
The bacterial diversity of cake layer in genus level.

Figure 2 illustrates the bacterial diversity of cake layer in genus level. The genus *Lentimicrobiaceae* was dominant, accounting for 8.47% in the cake layer. *Lentimicrobiaceae* belonging to *Bacteroidetes* was strictly anaerobic methane producing slow-growing bacteria [44]. Genus *Longilinea* was secondly dominant while genus *Trichococcus* was thirdly dominant accounting 7.50% and 6.49% respectively from the total abundance. *Trichococcus* was reported in anode biofilms [45], and on polypropylene filter media in a fixed biofilm reactor for wastewater treatment. The genus *Anaerolineaceae* (4.88%) containing carbohydrate-fermenting acetogenic filamentous bacteria was also recorded here [44]. Filamentous bacteria can more easily adhere to the membrane surface. However, in-depth analysis of bacterial composition in genus level in membrane cake layer was very limited in most studies.

#### 3.3.4. Top Archaea in Genus Level in Cake Layer

**Figure 3:**
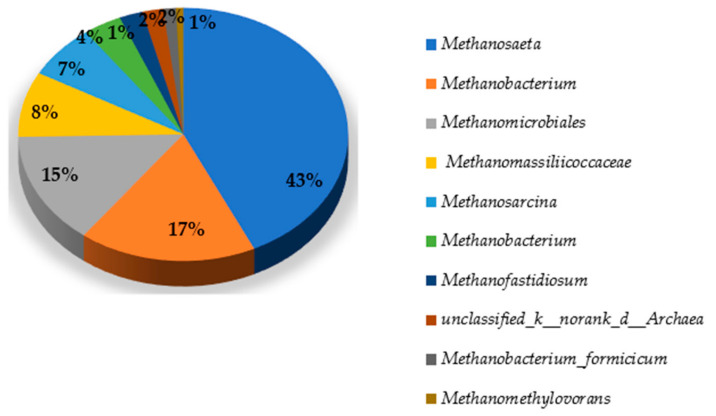
The cake layer archaeal diversity in Genus Level.

The Figure 3 shows the top archaea in genus level in the cake layer. The genus *Methanosaeta* (43%) was the most predominant in the cake layer. The acetoclastic *Methanosaeta* is not only filamentous but also they are aggregate microorganisms [43]. Thus, they might easily attach and grow in the cake layer. *Methanobacterium* genus (17.24%) was secondly dominant. *Methanobacterium* is a strict hydrogenotrophic methanogen using hydrogen and carbon dioxide to form methane [46] *Methanomicrobiales* (14.29%) was thirdly dominant. Subsequently, *Methanomassiliicoccoceae* (8.26%) and *Methanosarcina* (6.87%) were abundant. The genus *Methanosarcina* performs acetoclastic, hydrogenotrophic, and methylotrophic methanogenesis [46].

### 3.4. Comparison of the Bulk Sludge and the Cake Layer

#### 3.4.1. The Bacterial Diversity in Bulk Sludge Versus Cake Layers in Phylum Level

**Figure 4:**
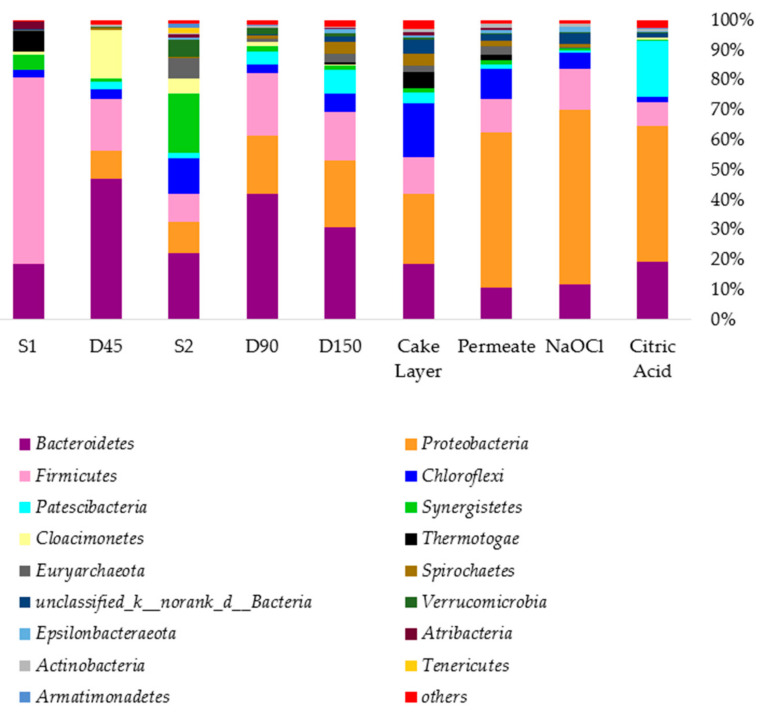
The community bar plot analysis of bacteria at bulk sludge, cake layer and the cleaning solutions in phylum.

Figure 4 shows the presence of different bacteria phyla in the bulk sludge, cake layer and the cleaning solutions. In this study two seed samples were used on Day 01 and 50 respectively, (i) from the Gao’antun wastewater reclamation plant in Beijing which is a partially hydrolyzed thermophilic sludge, (ii) anaerobic digester sludge from lab scale reactor treating potato starch wastewater at mesophilic conditions [11]. The first seed sludge was dominant with *Bacteroidetes* (18.6%), *Firmicutes* (62.3%), *Synergistetes* (4.96%) and *Thermotogae* (7.08%). The second seed sludge was dominant with *Bacteroidetes* (22.3%), *Proteobacteria* (10.19%), *Firmicutes* (9.60%), *Synergistetes* (19.8%), *Verrucomicrobia* (5.51%), and *Euryarchaeota* (6.94%). On Day 150, the bulk sludge was dominant with *Bacteroidetes* (30.92%), *Proteobacteria* (22.4%), *Firmicutes* (15.97%), *Chloroflexi* (6.10%), *Patescibateria* (7.92%), *Spirochaetes* (4.15%), *Synergistetes* (1.36%) [11]. The reactor operational conditions such as SRT, Hydraulic Retention Time (HRT), temperature evidently affect the microbial community development in the bulk sludge and membrane fouling. This was discussed in our previous work [11]. Here, the cake layer microbial community is shown in Figure 4. Remarkably, the cake layer bacterial community were very similar to those of the bulk sludge on Day 150. In some previous research, the microbial community in bulk sludge and membrane fouling layer were also similar in MBR systems treating municipal wastewater [1,24].

The genus level classification of bacterial community bar plot is illustrated in Figure S2. On day 150 in genus level the bulk sludge was dominant with *Trichococcus* (11.6%), *Lentimicrobbiaceae* (8.59%), *Chlorubium* (7.2%), *unclassified Patescibacteria* (6.48%), g-DMER64 (6.1%), *Aeromonas* (3.67%), *Spirochaetacea* (3.6%), *Rikellanaceae* (3.4%), *Aquaspirillum* (2.56%), no rank *Anaerolineaceae* (2.03%), *Burkholderiaceae* (1.0%). As mentioned in Section 3.3.3, the cake layer also contained *Lentimicrobiaceae* (8.47%), *Longilinea* (7.5%), *Trichococcus* (6.49%), *Anaerolineaceae* (4.8%) and *Rikellanaceae* (*1.29%*). *Trichococcus* and *Aeromonas* are generally found in sewage plants [41]. *Burkholderiaceae* is reported to have the ability to form biofilm and high survival in unsuitable environments [47]. Genus *Rikellanaceae* is an anaerobic and facultatively aerobic heterotrophic taxa [48]. The genus level bacterial community analysis also further revealed that in ambient temperature conditions the AnCMBR bacterial community dwelling in the cake layer of yttria ceramic tubular membrane was mostly similar to that of the bulk sludge. This is very important as it helps to predict the biofouling microbial community in the cake layer by investigating the bulk sludge community. Furthermore, Table 5 gives the alpha diversity indexes of the bacterial and archaeal community in bulk sludge, cake layer, and the cleaning solutions. The alpha diversity based on the Shannon and Simpsons index show that the cake layer microbial diversity was slightly higher than the bulk sludge community. The Shannon index for the cake layer and the bulk sludge was 4.48, 4.33 on Day 150, respectively. This might be due to the prolonged development of different bacterial communities in the membrane cake layer.

#### 3.4.2. Archaea Community in Bulk Sludge Versus Cake Layer

**Figure 5:**
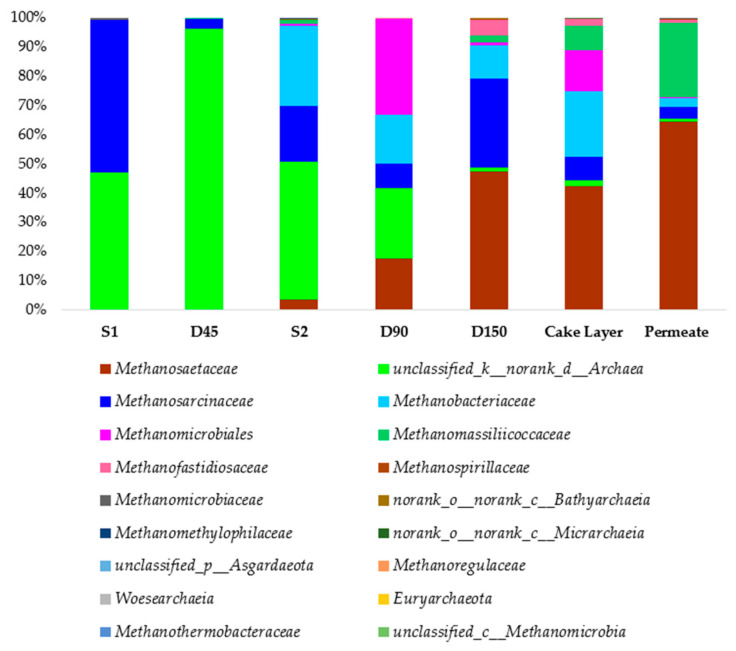
The community bar plot analysis of archaeal at family level sludge, cake layer and cleaning solution.

The Figure 5 depicts the archaea community at family level in the bulk sludge, cake layer and cleaning solutions. According to Day 150, the predominant archaea in family level in the anaerobic bulk sludge were *Methanosaetacea* (47.4%), *Methanosarcinaceae* (30.5%), *Methanobacteriaceae* (11.1%), *Methanofastidiosaceae* (5.35%), *Methanomassiliicoccacea* (2.3%), which were common methanogens. The special feature of the archaeal community denotes that the cake layer archaeal community was very similar to that of the bulk sludge community indicating the presence of similar archaeal families in the cake layer (i.e, *Methanosaetacea*, *Methanosarcinaceae* and *Methanobacteriacea*). However, the Simpson diversity index (1.56) on Day 150 (Table 5) was slightly less than that of the cake layer (1.81), indicating slightly high archaeal diversity on the surface of the membrane, which might be attributed to long term growth of respective archaea families on the membrane surface. Participation of these archaea families’ membrane fouling should be further studied in the future due to scarcity of previous studies.

### 3.5. The Presence of Bacteria and Archaeal in Cleaning Solutions

When the membrane is cleaned with different cleaning solutions, the bacteria and archaea developed on the membrane surface are cleaned based on the effectiveness of the cleaning solutions. Therefore, microbial community investigation of cleaning solutions helps to identify the dominant microbial community in the membrane cake layer. It also helps to decide suitable cleaning solutions and membrane fouling control approaches. According to Figure 5, in the permeate cleaning solution *Proteobacteria* was abundant (51.9%), then *Firmicutes* (11.29%), *Bacteroidetes* (10.65%) and *Chloroflexi* (9.80%) were abundant. The NaOCl cleaning solution also indicated more or less similar abundance with the permeate cleaning indicating *Proteobacteria* (58.31%), *Firmicutes* (13.84%) and *Bacteroidetes* (11.8%) as abundant bacteria. Those bacteria phyla were common to the citric acid cleaning solution while *Patescibacteria* (18.66%) were also abundant. In order to clearly define the contribution of different bacteria phyla to cake layer formation, a conceptual drawing was developed based on Choi et al., 2017 as presented in Supplementary Materials Figure S3. The cake layer was divided as inner and outer by Choi et al., 2017 [19]. In our previous publication it was termed as loosely attached cake layer and strongly attached cake layer based on Dong et al., 2015 [49]. In Figure S3 it was postulated that pore blocking microbial community is represented by citric acid solution, which could be the pioneers of the biofouling formation. The considerable presence of *Patescibacteria* over 5% abundance in citric acid cleaning indicated that they might have contributed initially to pore blocking. The outer cake layer can be easily removed by permeate cleaning and then the inner cake layer can be removed by NaOCl (The flux recovery values are indicated in the Table S1 after each cleaning process). NaOCl cleaning has given the highest 86% flux recovery. However, the bacteria contributing to pore blocking are the most difficult to remove.

Considering the archaeal diversity in the cleaning solutions, the results could be obtained only for permeate cleaning as the extracted DNA samples of citric acid and NaOCl cleaning solutions (less than 1 mL) were not sufficient for microbial community investigations. However, the permeate cleaning solution was abundant with *Methanosaetacea* (64.51%) and *Methanomassiliicoccaceae* (25.69%), which can be easily attached to the cake layer.

The alpha diversity indexes for cleaning solutions shown in Table 5 further indicate the effectiveness of cleaning solutions. Accordingly, Shannon and Sobs diversity indices show the highest values in permeate cleaning and lower values corresponding to NaOCl and citric acid cleaning. However, our previous study indicated that NaOCl cleaning solution has shown more microbial diversity [12]. Obviously, this discrepancy was due to changes in the operational conditions and cleaning protocols.

## 4. Conclusions

Cake layer has played a pivotal role in membrane fouling of yttria-based ceramic tubular membrane representing over 60% of the total membrane resistance. The cake layer microbial fouling community represented that of the anaerobic bulk sludge community of the AnCMBR for domestic wastewater treatment at the ambient conditions. The dominant bacteria were *Proteobacteria*, *Bacteroidetes, Firmicutes* and *Chloroflexi*, and the dominant archaea families were *Methanosaetacea* and *Methanobacteriaceae.* In the genus level of bacterial community, *Lentimicrobiaceae, Longilinea,* and *Trichococcus* were abundant in the cake layer. These bacteria and archaea phyla have played a major role in formation of biofouling of yttria-based ceramic tubular membrane. Therefore, these microbial phyla and genus were capable of surviving in the prevailed CFV in the ceramic yttria tubular membrane. This study provides the first evidence for the responsible microbial community for ceramic yttria-based tubular membrane fouling. This finding is important for future surface modification and development of antifouling membranes for AnCMBR.

## Figures and Tables

**Table 1:** The calculated values for *R_t_*, *R_m_*, *R_r_* and *R_ir_.*

Resistance	Value
*R_t_* (after 150 days operation)	8.09 × 10^12^
*R_m_*	2.02 × 10^12^
*R_r_*	5.64 × 10^12^
*R_ir_*	0.42 × 10^12^

**Table 2:** The comparison of the cake layer resistance at different membrane bioreactor (MBR) studies.

Reactor Type	Reactor Volume (L)	Wastewater Type	Membrane Module	Membrane Material	Operational Mode	Cake Layer Resistance (*R_r_*)	Fouling Control Method	Reference
AnCMBR	3.6	DWW	FS	Ceramic	--	95.2%	--	[9]
MBMBR	12.8	DWW	FS	Ceramic		84.8%/79.4%	bio carriers	[35]
UAGB	4	DWW	HF	PVDF		92%		[36]
CSTR	5	Synthetic DWW	HF	PVDF	8 min permeation 2 min relaxation	89–87.4%	hydrodynamic control	[33]
A/O MBR	3	tannery effluent	HF	PVC	suction mode of 10 min on/0.5 min off	80%	--	[34]
AnCMBR	15	DWW	T	Ceramic	AnCMBR	69%	DWW	This study

AnCMBR: anaerobic ceramic membrane bioreactor, MBMBR: moving bed membrane bioreactor, UAGB: upflow anaerobic granular bed, CSTR: continuous stirred tank reactor, A/O MBR: aerobic membrane bioreactor, DWW: domestic wastewater, FS: flat sheet, HF: hollow fiber, T: tubular, PVDF: Polyvinylidene fluoride, PVC: Polyvinyl chloride.

**Table 3:** The bacteria diversity of cake layer in phylum level.

Bacteria Phyla	Abundance (%)	Bacteria Phyla	Abundance (%)
*Proteobacteria*	23.37	*Euryarchaeota*	1.87
*Bacteroidetes*	18.83	*Synergistetes*	1.17
*Chloroflexi*	18.26	*Actinobacteria*	1.09
*Firmicutes*	12.02	*Atribacteria*	0.92
*Thermotogae*	5.49	*Epsilonbacteraeota*	0.81
*unclassified_k__norank_d__Bacteria*	4.96	*Verrucomicrobia*	0.54
*Spirochaetes*	3.93	*Cloacimonetes*	0.19
*Patescibacteria*	3.58	*Armatimonadetes*	0.09
*others*	2.79	*Tenericutes*	0.01

**Table 4:** The cake layer archaea diversity in the family Level.

Archea Family	Abundance (%)	Archea Family	Abundance (%)
*Methanosaetacea*	42.64	*Methanospirillaceae*	0.06
*Methanobacteriaceae*	22.40	*norank_o__norank_c__Micrarchaeia* *Methanomethylophilaceae*	0.05 0.03
*Methanomicrobiales*	14.29		
*Methanomassiliicoccaceae*	8.37	*unclassified_p__Asgardaeota*	0.03
*Methanosarcinaceae*	7.75	*norank_o__norank_c__Bathyarchaeia*	0.02
*Methanofastidiosaceae*	2.28	*Methanomicrobiaceae*	0.009
*unclassified_k__norank_d__Archaea*	2.01	*Methanoregulaceae*	0.001

**Table 5:** The alpha diversity index for bacteria and archaeal in cleaning solutions.

Sample\Estimators	Sobs	Shannon	Simpson	Ace	Chao	Coverage
S1	291	3.462506	0.05852	303.9668	304	0.999618
S2	664	4.245772	0.029664	779.7667	786.7662	0.997938
D45	392	2.778037	0.182463	477.9779	502.5349	0.998259
D90	685	3.876093	0.051158	880.5341	895.45	0.997332
D150	768	4.338893	0.034709	959.76	997.2	0.997129
Cake layer	908	4.844364	0.018429	1081.579	1086.443	0.997169
Permeate	838	4.489069	0.029975	1073.885	1079.606	0.99488
NaOCl	789	4.225402	0.039877	1007.753	989.4492	0.994787
Citric acid	1092	4.18902	0.050877	1190.10	1164.675	0.996111
Archaeal diversity						
**Sample\Estimators**	**Sobs**	**Shannon**	**Simpson**	**Ace**	**Chao**	**Coverage**
S1	181	2.060121	0.278739	196.9229	191.3448	0.999497
S2	435	3.234795	0.084348	474.861	462.8088	0.999022
D45	224	1.357059	0.509039	314.5664	310.0588	0.997753
D90	110	2.260526	0.168098	132.8407	131.4286	0.999592
D150	81	1.562673	0.315953	137.2779	97.25	0.999607
Cake layer	107	1.819329	0.245727	190.9432	157.2143	0.999254
Permeate	96	1.095714	0.483232	127.0463	132.25	0.999545

## Supplementary Materials

The following are available online at https://www.mdpi.com/2077-0375/11/2/108/s1, Figure S1: The schematic diagram of the reactor setup, Figure S2: The bacterial community bar plot analysis at genus level, Figure S3: Conceptual visualization of yttria-based tubular ceramic membrane fouling bacterial diversity, Table S1: The membrane flux recovery of cleaning solutions.
