# Supplementary material for: New Insights into the Microbial Diversity of Cake Layer in Yttria Composite Ceramic Tubular Membrane in an Anaerobic Membrane Bioreactor (AnMBR)
Source: Membranes (Basel). 2021 Feb 3;11(2):108. doi: 10.3390/membranes11020108 (PMC7913466; doi:10.3390/membranes11020108)
Supplement: Supplementary file 1 [file membranes-11-00108-s001.pdf]

membranes

MDPI

Article

New Insights into the Microbial Diversity of Cake Layer in Yttria Composite Ceramic Tubular Membrane in an Anaerobic Membrane Bioreactor (AnMBR)

Rathmalgodage Thejani Nilusha [1,2,3,4] and Yuansong Wei [1,3,4,5,*]

[1] State Key Joint Laboratory of Environmental Simulation and Pollution Control, Research Center for Eco-Environmental Sciences, Chinese Academy of Sciences, Beijing 100085, China; nthejani@yahoo.com
[2] Environment Technology Section, Industrial Technology Institute, 363, Bauddhaloka Mawatha, Colombo 07 00700, Sri Lanka; thejani@iti.lk
[3] Department of Water Pollution Control Technology, Research Center for Eco-Environmental Sciences, Chinese Academy of Sciences, Beijing 100085, China
[4] University of Chinese Academy of Sciences, Beijing 100049, China
[5] Institute of Energy, Jiangxi Academy of Sciences, Nanchang 330029, China
* Correspondence: yswei@rcees.ac.cn; Tel.: +86-10-6284-9690

Citation: Nilusha, R.T.; Wei, Y. New Insights into Microbial Diversity of Cake Layer in Yttria Composite Ceramic Tubular Membrane in an Anaerobic Membrane Bioreactor (AnMBR). *Membranes* **2021**, *11*, 108. https://doi.org/10.3390/membranes11020108

Academic Editor: Amine Charfi
Received: 23 December 2020
Accepted: 27 January 2021
Published: 3 February 2021

Publisher's Note: MDPI stays neutral with regard to jurisdictional claims in published maps and institutional affiliations.

Copyright: © 2021 by the authors. Licensee MDPI, Basel, Switzerland. This article is an open access article distributed under the terms and conditions of the Creative Commons Attribution (CC BY) license (http://creativecommons.org/licenses/by/4.0/).

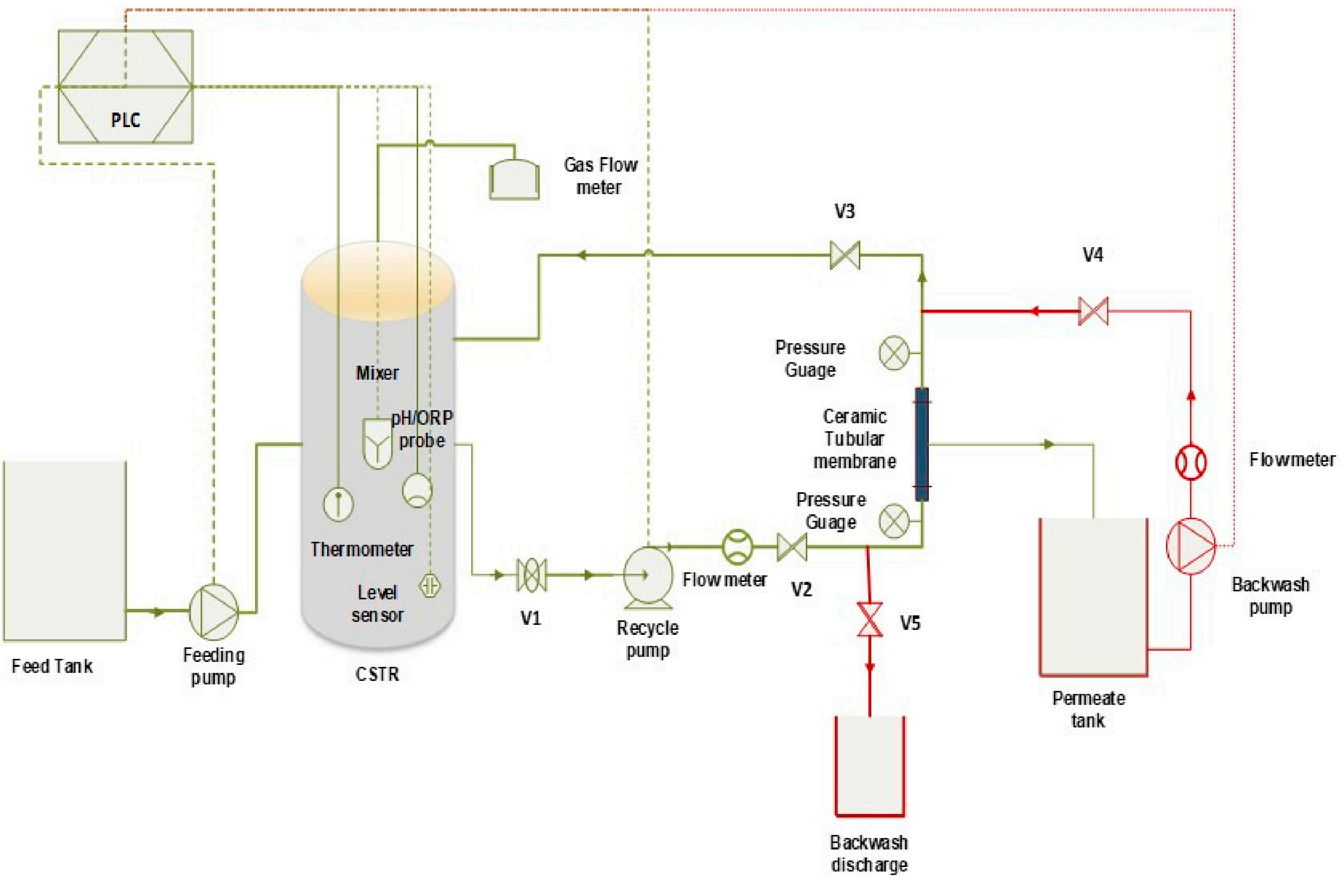

Figure S1. The Schematic diagram of the reactor setup.

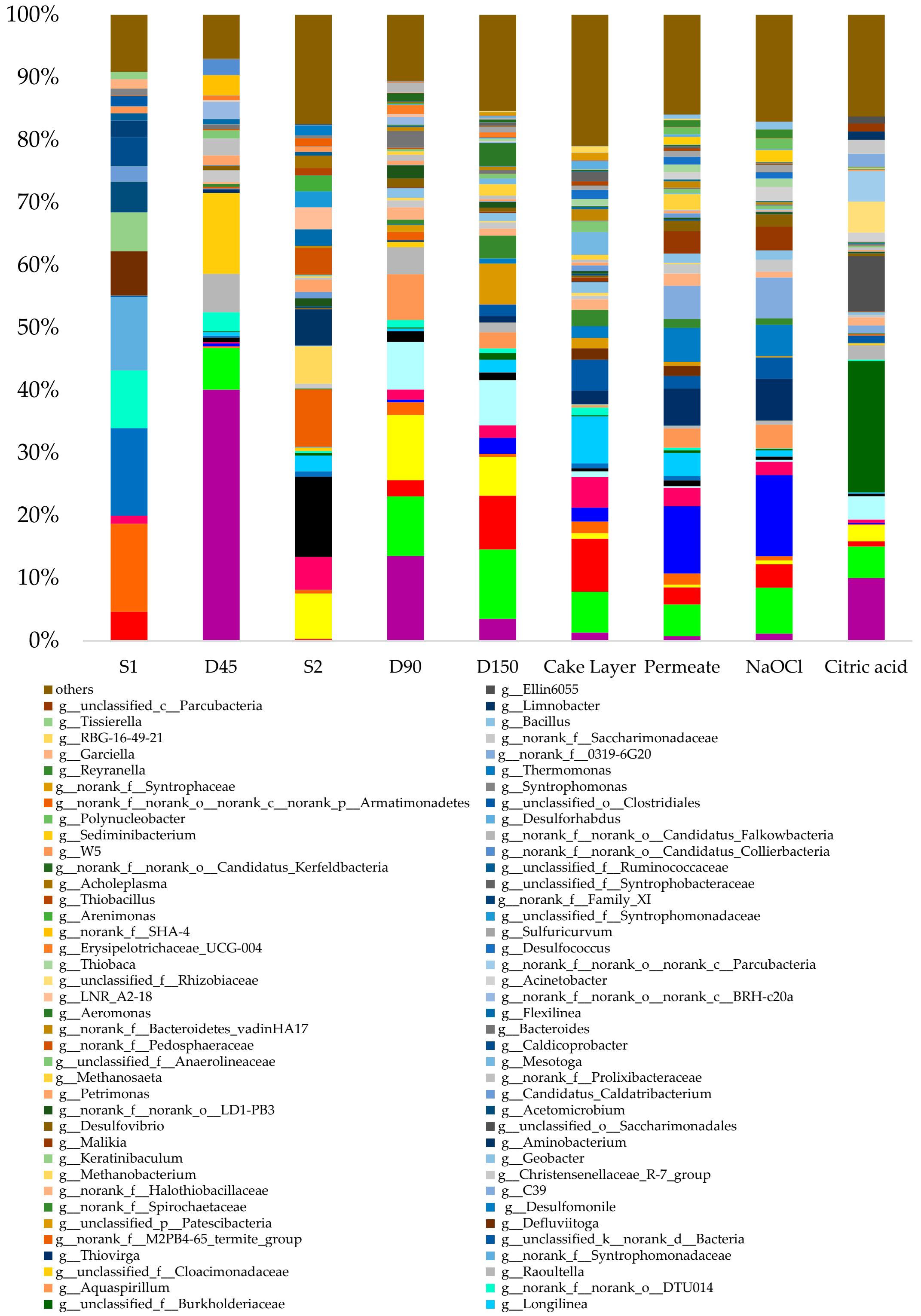

Figure S2. The microbial community bar plot analysis at genus level.

Outer Cake Layer (permeate cleaning)	Inner Cake Layer (NaOCl cleaning)	Pore blocking (Citric acid cleaning)
Bacteroidetes (10.65%)	*Bacteroidetes (11.80%)*	*Bacteroidetes (19.41%)*
Proteobacteria (51.96)	*Proteobacteria (58.31%)*	*Proteobacteria (45.20%)*
Firmicutes (11.29%)	*Firmicutes (13.84%)*	*Firmicutes (7.97%)*
Choroflexi (9.80%)	*Choroflexi (5.19%)*	*Choroflexi (1.91%)*
Spirochaetes (1.94%)	*Spirochaetes (1.16%)*	*Spirochaetes (0.29%)*
Actinobacteria (1.19%)	*Actinobacteria (1.10%)*	*Actinobacteria (0.92%)*
Thermotogae (1.78%)		*Pastescibacteria (0.84%)*
Atribacteria (0.69%)		
Synergistetes (1.32%)		
Euryarcheota (2.84%)		
Pastescibacteria (1.69%)		

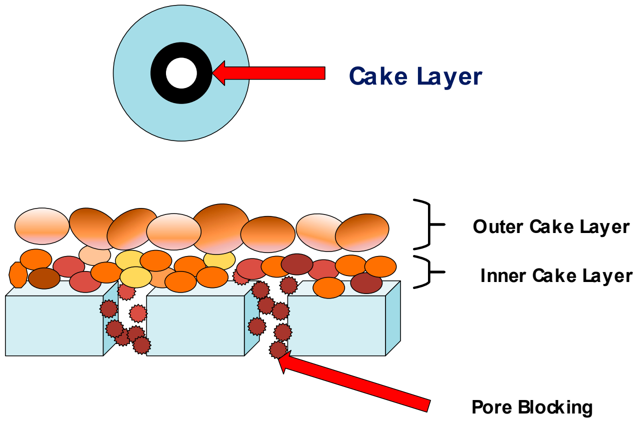

Figure S3. Conceptual visualization of yttria-based tubular ceramic membrane fouling bacterial diversity.

Table S1. The membrane flux recovery of cleaning solutions.

Parameter	Flux (LMH)	Flux recovery
Virgin membrane	234.50	--
Fouled membrane	16.36	--
After permeate cleaning	99	7%
NaOCl cleaning	218.18	86%
Citric acid cleaning	135.45	57.5%
